# Enhancing effect of natural adjuvant, panduratin A, on antibacterial activity of colistin against multidrug-resistant *Acinetobacter baumannii*

**DOI:** 10.1038/s41598-024-60627-0

**Published:** 2024-04-29

**Authors:** Nalumon Thadtapong, Soraya Chaturongakul, Chanita Napaswad, Padungsri Dubbs, Sunhapas Soodvilai

**Affiliations:** 1 Research Center of Transport Protein for Medical Innovation, Department of Physiology, Faculty of Science, Mahidol University, Bangkok, Thailand; 2 Center for Advanced Therapeutics, Institute of Molecular Biosciences, Mahidol University, Nakhon Pathom, Thailand; 3 Department of Microbiology, Faculty of Science, Mahidol University, Bangkok, Thailand; 4 Excellent Center for Drug Discovery, Mahidol University, Bangkok, Thailand

**Keywords:** *Acinetobacter baumannii*, Colistin, Multidrug resistance, Panduratin A, Adjuvant, Drug discovery, Microbiology

## Abstract

Colistin- and carbapenem-resistant *Acinetobacter baumannii* is a serious multidrug resistant (MDR) bacterium in clinical settings. Discovery of new antibacterial drugs against MDR is facing multiple challenges in drug development. Combination of known antibiotics with a robust adjuvant might be an alternative effective strategy for MDR treatment. In the study herein, we report an antibiotic adjuvant activity of a natural compound panduratin A from fingerroot (*Boesenbergia rotunda*) as a potent adjuvant to colistin. The present study investigated the antibiotic adjuvant effect of panduratin A against 10 colistin- and carbapenem-resistant *A. baumannii*. Antibacterial activities were tested by broth microdilution method. Biofilm assay was used to determine the efficacy of panduratin A in biofilm formation inhibition on two representative strains Aci46 and Aci44. Genomic and transcriptomic analyses of colistin- and carbapenem-resistant *A. baumannii* strains were used to identify potential resistance and tolerance mechanism in the bacteria. Panduratin A-colistin combination showed an increased effect on antibacterial in the *A. baumannii*. However, panduratin A did not improve the antibacterial activity of imipenem. In addition, panduratin A improves anti-biofilm activity of colistin against Aci44 and Aci46, the colistin- and carbapenem-resistant *A. baumannii*. Panduratin A markedly enhances bactericidal and anti-biofilm activity of colistin against colistin- resistant *A. baumannii*. Based on genome comparisons, single nucleotide polymorphism (SNP) patterns in six genes encoding biofilm and lipid A biosynthesis were shared in Aci44 and Aci46. In Aci44, we identified a partial sequence of *pmrB* encoding a polymyxin resistant component PmrB, whereas a full length of *pmrB* was observed in Aci46. RNA-seq analyses of Aci44 revealed that panduratin A-colistin combination induced expression of ribosomal proteins and oxidative stress response proteins, whereas iron transporter and MFS-type transporter systems were suppressed. Panduratin A-colistin combination could promote intracellular reactive oxygen species (ROS) accumulation could lead to the cidal effect on colistin-resistant *A. baumannii*. Combination of panduratin A and colistin showed a significant increase in colistin efficacy against colistin- resistant *A. baumannii* in comparison of colistin alone. Genomic comparison between Aci44 and Aci46 showed mutations and SNPs that might affect different phenotypes. Additionally, based on RNA-Seq, panduratin A-colistin combination could lead to ROS production and accumulation. These findings confirmed the potency of panduratin as colistin adjuvant against multidrug resistant *A. baumannii*.

## Introduction

Emerging and persistent multidrug resistant (MDR) bacteria pose challenging problems to One Health. The last-line drugs used in humans such as carbapenems and colistin are used against severe Gram-negative multidrug bacterial infection^1^. Carbapenems such as imipenem and meropenem are potential drugs in a beta-lactam group that inhibit the bacterial cell wall synthesis, specifically transpeptidation^2^. Inhibition of peptidoglycan crosslinking leads to cell lysis and cell death^2^. However, carbapenem-resistant *Acinetobacter baumannii* was discovered^3^ and, in 2017, WHO categorized carbapenem-resistant *A. baumannii* (CRAB) as one of priority 1 (critical tier) pathogen list of antibiotic-resistant bacteria^4^. Colistin (polymyxin E) is one of polymyxins that targets lipid A in lipopolysaccharide and disrupts the outer membrane leading to cytoplasm leakage^5^. Colistin is considered to be an alternative drug for treating CRAB^6^. Unfortunately, CRAB that resists to colistin was also discovered^7,8^. Therefore, discoveries of effective therapeutic approaches are critically required.

Natural products have gained tremendous attention as alternative compounds for solving the antibiotic resistance problems in bacteria^9^. Some natural compounds are candidates in eliminations of MDR bacterial infection. Panduratin A is a natural compound extracted from fingerroot (*Boesenbergia rotunda*)^10^. Panduratin A alone has antimicrobial activities against Gram-positive bacteria such as *Staphylococcus* spp. and *Enterococci* clinical isolates^11,12^ and anti-biofilm activities against *Streptococcus mutans*, *Streptococcus sanguis* and *Actinomyces viscosus*^13^, but not against Gram-negative bacteria^14^. Panduratin A has also been demonstrated to have an anti-SARS-CoV-2 property^15^.

Our recent study demonstrated that panduratin A has a nephroprotective property against colistin-induced renal injury^14^. Interestingly, the compound did interfere with efficacy of the antibiotic against a reference strain of colistin-susceptible *Escherichia coli* by decreasing MIC and MBC values, suggesting a potential use of panduratin A in combination with nephrotoxic antibiotics^14^. The present study investigated whether panduratin A could act as an adjuvant to improve the antibacterial activity of colistin and carbapenem against colistin- and carbapenem-resistant *A. baumannii*.

## Methods

### Bacterial strains

Ten colistin-resistant^16^ and one colistin susceptible (ATCC 19606) *A. baumannii* (Table 1), used for colistin susceptibility tests in the presence and absence of panduratin A, were from the stock cultures kept at − 80 °C in 10% Difco™ skim milk (Becton and Dickinson, USA) containing 10% glycerol.

**Table 1:** The MIC and MBC of panduratin A (PanA in mg/L) in combination with colistin (Col) or imipenem (Imi) in multidrug-resistant *A. baumannii.*

Isolates	Col MIC/MBC	Col + 5 µM PanA MIC/MBC	Col + 2.5 µM PanA MIC/MBC	Col + 1 µM PanA MIC/MBC	Imi MIC/MBC	Imi + 5 µM PanA MIC/MBC	Imi + 2.5 µM PanA MIC/MBC	Imi + 1 µM PanA MIC/MBC
Aci9	64/64	2/2	4/4	4/4	32/64	32/> 64	32/64	64/64
Aci13	64/64	2/2	2–4/2–4	4/4	> 64/> 64	64/> 64	64/> 64	64/> 64
Aci31	16/16	2/2	2/2	4/8	> 64/> 64	64/64	64/64	> 64/> 64
Aci32	16/16	2/2	2/2	8/8	> 64/> 64	64/64	64/> 64	64/64
Aci33	16/16	2/2	2/2	4/4	64/> 64	> 64/> 64	64/64	64/64
Aci34	64/64	2–4/2–4	8/8	8/8	64/64	64/> 64	64/64	64/64
Aci35	16/16	1–2/1–2	2/2	8/8	64/> 64	64/> 64	64/> 64	64/> 64
Aci36	16/16	2/2	2/2	4/4	> 64/> 64	64/> 64	64/64	64/64
Aci44	64–128/64–128	1–2/1–2	4/4	64/64	> 256	> 256	> 256	> 256
Aci46	> 1024/> 1024	16–32/16–32	64/64	> 1024/> 1024	> 256	> 256	> 256	> 256
ATCC 19606	1/1	1/1	1/1	1/1	< 0.5/< 0.5	< 0.5/< 0.5	< 0.5/< 0.5	< 0.5/< 0.5

The results are ranges of MIC and MBC. The experiments were tested in three biological replicates.

### Panduratin A

Rhizomes of *Boesenbergia rotunda* were dried, finely grounded, and successively percolated with 95% ethanol for three days at room temperature. The ethanolic extract was concentrated and lyophilized to yield a crude extract. Panduratin A was isolated from the extract by our previously described method^17^. The purity of panduratin A was determined by High Perform Liquid Chromatography (HPLC) analysis (Supplementary Fig. S1). Panduratin A with the purity of 98% was used in this study.

### Antimicrobial susceptibility test by broth microdilution assay

Antibiotic susceptibilities were determined by broth microdilution method performed according to the Clinical and Laboratory Standards Institute (CLSI) guideline^18,19^ for colistin, imipenem, meropenem, panduratin A, and panduratin A-antibiotics combinations. Aci44 and Aci46 were streaked on Difco™ Mueller Hinton agar (MHA, Becton and Dickinson, USA) and incubated overnight at 37 °C. *E. coli* ATCC 25922 and *A. baumannii* ATCC 19606 were used as control reference strains. Single colonies were picked and resuspended in normal saline solution (0.85% NaCl) and adjusted to the optical density at 600 nm (OD_600_) to 0.08–0.12 to obtain approximately 1 × 10^8^ colony forming units (CFU)/mL. The cell suspension was diluted for 100-folds to prepare 1 × 10^6^ CFU/mL in Difco™ cation-adjusted Mueller Hinton broth (CAMHB, Becton and Dickinson, USA). Antibiotic concentrations used in this study ranged from 0.5 to 1024 mg/L and 0.125–256 mg/L for colistin and imipenem, respectively. As for drug-adjuvant combinations, panduratin A was added to each concentration of antibiotics to obtain a final concentration of either 5, 2.5, and 1 µM. A total volume of 200 µL was obtained using a mixture of 100 µL of antibiotic alone or in combination with panduratin A and 100 µL of cell suspension. After incubation at 37 °C for 20–24 h, the minimal inhibitory concentration (MIC) was observed and cell viability was measured by MTT staining^20^. The absorbance of formazan dissolution was detected at 570 nm using a microplate reader (Azure Ao Absorbance Microplate Reader, Azure Biosystems, USA). Relative OD was calculated by dividing OD_570_ of drug-contained wells with that of drug-free wells^21^. The relative OD_570_ of 0.1 was the cut-off for no detection. The minimal bactericidal concentration (MBC) was determined by sub-culturing tubes containing drug concentrations ≥ MIC onto MHA. The plates were incubated at 37 °C for 24 h for colony count. The MBC was defined as the lowest concentration of drugs or combined drugs which produced > 99.9% reduction as compared to initial inoculum, according to CLSI^18^. MIC and MBC were performed from three individual replicates. For an antibiotic, bactericidal effect is when MBC/MIC ratio is ≤ 4 and bacteriostatic effect is when MBC/MIC is > 4^22^.

### Determination of colistin enhancing activity by panduratin A

The antimicrobial effects of panduratin A-colistin combinations were evaluated by MBC/MIC ratio, fractional inhibitory concentration (FIC), and fractional bactericidal concentration (FBC). Since panduratin A did not affect growth of Aci44 and Aci66, FIC and FBC were only calculated for colistin using the equations below:

$$FIC \;of \;colistin =\frac{MIC \;of \;PanA+colistin}{MIC \;of \;colistin \;alone}$$

$$FBC \;of \;colistin=\frac{MBC \;of \;PanA+colistin}{MBC \;of \;colistin \;alone}$$

The interaction between panduratin and colistin was defined as enhanced when FIC and FBC ≤ 0.5, no interaction when FIC and FBC > 0.5–4, and antagonism when FIC and FBC ≥ 4. The criteria of definition was similar to FIC index and FBC index^23^. The calculation of FBC was same way by replacing MIC values with MBC values.

### Anti-biofilm formation assessed by MTT and crystal violet staining

Four different concentrations of colistin (Aci44: 1, 0.5, 0.25, 0.125 mg/L and Aci46: 16, 8, 4, 2 mg/L) and imipenem (16 mg/L) with three different concentrations of panduratin A (5, 2.5, 1 µM) were prepared for biofilm detection as compared to antibiotics alone and panduratin A alone. Maximum concentrations of colistin in anti-biofilm testing were 0.5xMIC colistin in combination with 5 µM panduratin A. Briefly, antibiotics and panduratin A-antibiotic combinations at varying concentrations were prepared. Each well was inoculated with Aci44 or Aci46 (final cell concentration of 5 × 10^5^ CFU/mL) in 200 µL total volume. The cultured 96-well plates were incubated at 37 °C for 24 h. After incubation, 100 µL of supernatant was taken from one replicate to determine cell viability by MTT staining^20^. The rest of culture broth was removed and washed with PBS twice to remove pellicle cells and floating cells. The biofilm was then stained by MTT^20^. For crystal violet staining^24^, culture was removed, and biofilm mass was washed and fixed by adding 200 µL of methanol for 15 min. After that, biofilm mass was dried at room temperature and 0.1% crystal violet (Sigma-Aldrich, USA) (200 µL) was added and incubated on benchtop for 30 min. The biofilm mass was washed twice with PBS and dissolved in 33% acetic acid (Merck, Germany) (200 µL). The total biomass in each well was quantified by detecting at 570 nm using microplate reader.

### Statistical analysis

All microbiological tests were presented in three biological replicates. All multiple comparisons were analyzed using one-way ANOVA with Tukey post-hoc test on PASW statistics 18. The P-value of < 0.05 considered as significant difference.

### Genomic DNA extraction and whole genome sequencing

Whole genomic DNA (gDNA) of Aci44 was extracted by modified Marmur procedure^8,25^. Briefly, Aci44 was inoculated in CAMHB and incubated at 37 °C, 200 rpm, overnight. After incubation, Aci44 (3 mL) was harvested and lysed by 10 mg/mL lysozyme (Geneaid, Taiwan). RNA and protein were removed using 0.6 mg/mL RNase A (Serva, Germany) and 0.1 mg/mL proteinase K (Geneaid, Taiwan), respectively. DNA was purified using phenol–chloroform extraction. Quality and quantity of gDNA were determined by 1% agarose gel electrophoresis (Bio-rad, USA), UV spectrophotometer (OD_260/280_, OD_260/230_ ratio) (DeNovix DS-11 FX + spectrophotometer, DeNovix, USA), Qubit dsDNA BR assay kit (Invitrogen, USA), and Bioanalyzer using a high-sensitive DNA chip (Agilent, USA). One hundred ng of extracted DNA was used for library preparation using Ovation Ultralow System V2 (Nugen, Switzerland) and sequenced on NovaSeq (Illumina, USA) for short-read sequencing. Nanopore platform was applied for long-read sequencing. One µg of gDNA was used for library preparation by Ligation Sequencing kit (SQK-LSK110, Oxford Nanopore, UK) and sequenced on MinION Nanopore (Oxford Nanopore, UK).

### Genome assembly and annotation

Raw reads from short-read and long-read sequencings were trimmed by Trim-Galore version 0.6.3^26^ and Porechop version 0.2.4^27^, respectively. The qualities of short-reads and long-reads were checked by FastQC version 0.11.9^28^ and Nanoplot version 1.28.2^29^, respectively. Trimmed reads from both platforms were hybrid assembled using Unicycler version 0.4.8^30^ and error corrected by Pilon version 1.23^31^. Genome coverage was calculated by SAMTools version 1.3^32^ in BV-BRC version 3.30.19^33^. The quality of assembled contigs was analyzed by QUAST version 5.0.2^34^ and visualized by Bandage version 0.8.1^35^. Coding sequences and functional genes were annotated by RASTtk version 1.3.0^36^. Identification of *A. baumannii* was confirmed by ribosomal multi-locus sequence typing (rMLST)^37^. Antibiotic resistance genes were predicted based on ResFinder 4.1^38^, NADRO^39^, and CARD^40^ databases. The *pmrB* genes from Aci44, Aci46, and ATCC 17978 were aligned together by Clustal Omega in EMBL-EBI^41^.

### Comparative genome and pairwise SNP analysis

Genome data from *A. baumannii* ATCC 17978 (drug sensitive, accession number: CP000521.1)^42^, Aci44 (colistin and carbapenem-resistance, accession number: CP101653), and Aci46 (colistin and carbapenem-resistance, accession number: JAHPZP000000000.1)^8^ were compared by Protein Family Sorter with PATRIC genus-specific families (PLfam) strategy in BV-BRC version 3.30.19^33^. Pairwise SNP analyses of Aci44 vs. ATCC 17978 and Aci44 vs. Aci46 were predicted by variation analysis mode in BV-BRC version 3.30.19^33^. Genome data of Aci44 were aligned to ATCC 17978 and Aci46 using BWA-mem alignment^43^ and SNP calling by FreeBayes^44^.

### RNA extraction and transcriptomic sequencing

Aci44 was cultured in CAMHB, incubated at 37 °C, until OD_600_ of 0.4–0.6 (mid-log phase) and diluted until OD_600_ of 0.08–0.12 (approximately 10^8^ CFU/mL). Cell suspension was diluted 100 folds in four different conditions [CAMHB, 0.1 mg/L colistin, 5 µM panduratin A, and combined 0.1 mg/L colistin and 5 µM panduratin A] and incubated at 37 °C, 200 rpm for 24 h. Bacterial transcriptional processes were stopped by adding 0.1 volume of stop solution (10% acid phenol pH 6.6 in absolute ethanol) and collected by centrifugation. Cell pellets were stored at − 80 °C. RNA was extracted from 3 mL of sample by RNeasy micro kit (Qiagen, Germany) and gDNA was eliminated by RNase-free DNase set (Qiagen, Germany). Quality and quantity of extracted RNA were measured by Nanodrop 1000 UV visible spectrophotometer (Thermo Scientific, USA) and TapeStation High Sensitivity RNA ScreenTape (Agilent, USA). One hundred ng of extracted total RNA was used to prepare the cDNA library, and ribosomal RNAs were removed using Universal Prokaryotic RNA-Seq Library Preparation kit (Tecan, Switzerland). The prepared cDNA libraries were sequenced on the two-channel sequencing by synthesis chemistry by Illumina NovaSeq platform (Illumina, USA).

### Transcriptomic analysis

Raw data of RNA-Seq were uploaded to BV-BRC version 3.30.19^33^ for analysis and the complete genome of Aci44 was used as a reference genome. Briefly, raw reads were trimmed by Trim Galore version 0.6.3^26^ and quality-checked by MultiQC^45^. Differential expressions were analyzed with Tuxedo strategy^46^. Trimmed reads were mapped with reference genome by Bowtie 2 version 2.5.1^47^ and mapped reads were measured by SAMTool^32^. Mapped reads were assembled, merged, and analyzed for differential expression using Cufflinks^46^, Cuffmerge^46^, and Cuffdiff^48^, respectively. Expression values were represented in FPKM (Fragments Per Kilobase transcript per Million mapped reads) and the fold changes of expression were calculated in log-ratio with two-fold change was set as a threshold. The q-value less than 0.05 was considered as significant difference. The functional genes and systems were annotated by RASTtk version 1.3.0^36^ in BV-BRC version 3.30.19^33^.

### Measurement of ROS production

The productions of reactive oxygen species (ROS) by Aci44 and Aci46 after treatment with colistin, panduratin A, or panduratin-colistin combination were measured using 2′-7′-Dichlorofluorescein (DCF) (Sigma-Aldrich, USA)^49^. Aci44 and Aci46 at 10^6^ CFU/mL in PBS were pre-incubated for 30 min with 5 µM DCF at 37 °C, then treated with colistin, panduratin A, or panduratin-colistin combination. Aci44 and Aci46 were treated with four conditions: (1) 0.05 × MIC (0.1 mg/L colistin for Aci44 and 1.6 mg/L colistin for Aci46) colistin combined with 5 µM panduratin A, (2) colistin alone, (3) 5 µM panduratin A alone, and (4) untreated (or CAMHB). The treated strains were incubated at 37 °C for 1 h in 96-black well plate. Twenty µM H_2_O_2_ treatment was used as a positive control. Fluorescence intensity was measured using Clariostar plus microplate reader (BMG Labtech, Germany) at excitation wavelength of 480 nm and emission wavelength of 530 nm.

## Results

### Panduratin A increases antibiotic activity of colistin against clinical isolation of *A. baumannii*

The antibiotic activities of panduratin A against 10 colistin-resistant *A. baumannii* were initially tested. The minimum inhibitory concentration (MIC) and minimum bactericidal concentration (MBC) values of colistin, colistin plus 2.5 µM panduratin A, and colistin plus 5 µM panduratin A were determined. As shown in Table 1, all clinical isolates resisted to colistin, as shown by the MIC values higher than 16 mg/L. The control strain ATCC 19606 was non-resistant to colistin. Combination of colistin and panduratin A at 5 µM significantly improved the sensitivity of colistin against *A. baumannii* by reducing the MIC and MBC of colistin.

**Figure 1:**
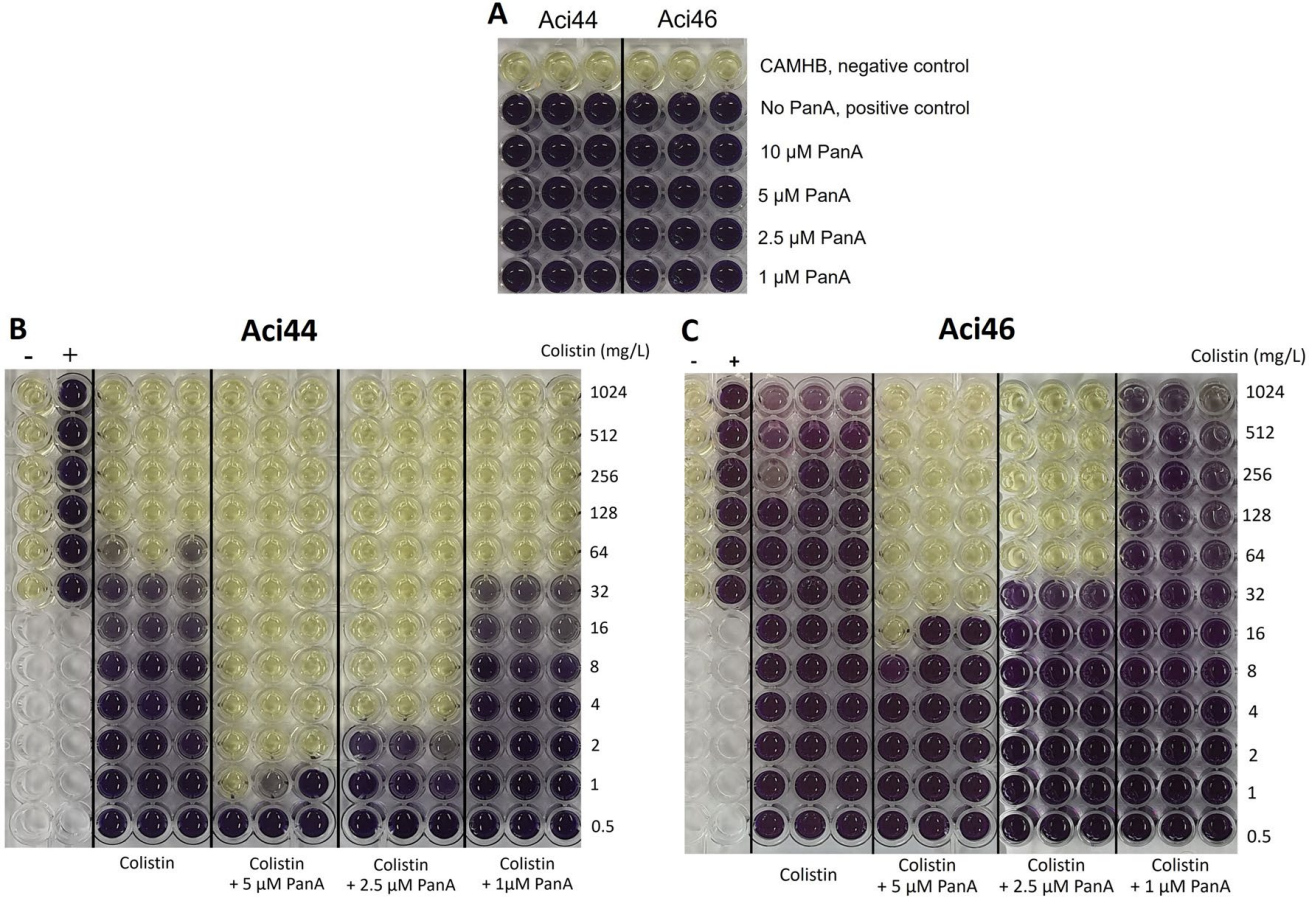
Evaluation of antibiotic activity of panduratin A (PanA) on *A. baumannii* Aci44 and Aci46. (**A**) cell viabilities of Aci44 and Aci46 in four different panduratin A concentrations (0 [or no PanA], 1, 2.5, 5, and 10 µM) were observed by MTT-staining. Uninoculated CAMHB cultures were used as negative controls. MIC determination of colistin alone and combined compounds against Aci44 (**B**) and Aci46 (**C**) using microdilution assay and MTT staining. Viable cells stained purple while dead cells were not stained and shown in yellow. The experiments were tested in three biological replicates.

Aci44 and Aci46 with the highest MIC and MBC values were selected for the subsequent studies. Treatment with panduratin A alone cannot eliminate Aci44 and Aci46 as shown by similar levels of alive cells in panduratin A treatments and control (Fig. 1A). Next, we determined whether panduratin A had an adjuvant activity based on our previous study which suggested its nephroprotective potency as an addition to colistin treatment^14^. Aci44 and Aci46 were treated with vehicle (DMSO), panduratin A alone, last-line drug alone (i.e., colistin and imipenem), and panduratin A-antibiotic combinations. The MIC and MBC values obtained from the combination of colistin and panduratin A at 1 µM was the same with colistin alone. Increase in concentrations of panduratin A to 2.5 and 5 µM significantly reduced the MIC values in a concentration-dependent manner compared with colistin alone (Fig. 1B,C and Table 1). At 5 µM, panduratin A was able to reduce the MICs by at least 32 folds from colistin alone to panduratin A -colistin combination. However, an increase in the concentration of panduratin A to 10 µM did not show a pronounced effect on colistin’s sensitivity (Supplementary Fig. S2). The MBC value in each condition was the same with MIC in both Aci44 and Aci46 (Table 1) indicating panduratin A could enhance the bactericidal activity of colistin. Combination of imipenem and panduratin A, however, did not affect viability of Aci44 and Aci46 (Table 1). To determine whether panduratin A had the enhancing effects on colistin, the interaction of panduratin A and colistin was analyzed by FIC and FBC calculation. As shown in Table 2, panduratin A at 2.5 and 5 µM in combination with colistin could enhance colistin activities confirming that panduratin A could be an adjuvant to colistin.

**Table 2:** Effects of colistin alone and panduratin A (PanA)-colistin combinations on Aci44 and Aci46.

Conditions	Aci44		Aci46	
	MBC/MIC	Interpretation	MBC/MIC	Interpretation
Colistin alone	1.33 ± 0.58	Bactericidal	1.00 ± 0.00	Bactericidal
Colistin + 5 µM PanA	1.00 ± 0.00	Bactericidal	1.00 ± 0.00	Bactericidal
Colistin + 2.5 µM PanA	1.00 ± 0.00	Bactericidal	1.00 ± 0.00	Bactericidal
Colistin + 1 µM PanA	1.00 ± 0.00	Bactericidal	1.00 ± 0.00	Bactericidal

Conditions	FIC	Interpretation	FIC	Interpretation
Colistin + 5 µM PanA	0.02 ± 0.01	Enhanced	0.26 ± 0.01	Enhanced
Colistin + 2.5 µM PanA	0.05 ± 0.02	Enhanced	0.06 ± 0.00	Enhanced
Colistin + 1 µM PanA	0.83 ± 0.29	No interaction	1.00 ± 0.00	No interaction

Conditions	FBC	Interpretation	FBC	Interpretation
Colistin + 5 µM PanA	0.02 ± 0.01	Enhanced	0.03 ± 0.01	Enhanced
Colistin + 2.5 µM PanA	0.04 ± 0.02	Enhanced	0.06 ± 0.00	Enhanced
Colistin + 1 µM PanA	0.67 ± 0.29	No interaction	1.00 ± 0.00	No interaction

The experiments were tested in three biological replicates.

### Anti-biofilm activities of panduratin A-colistin combination

**Figure 2:**
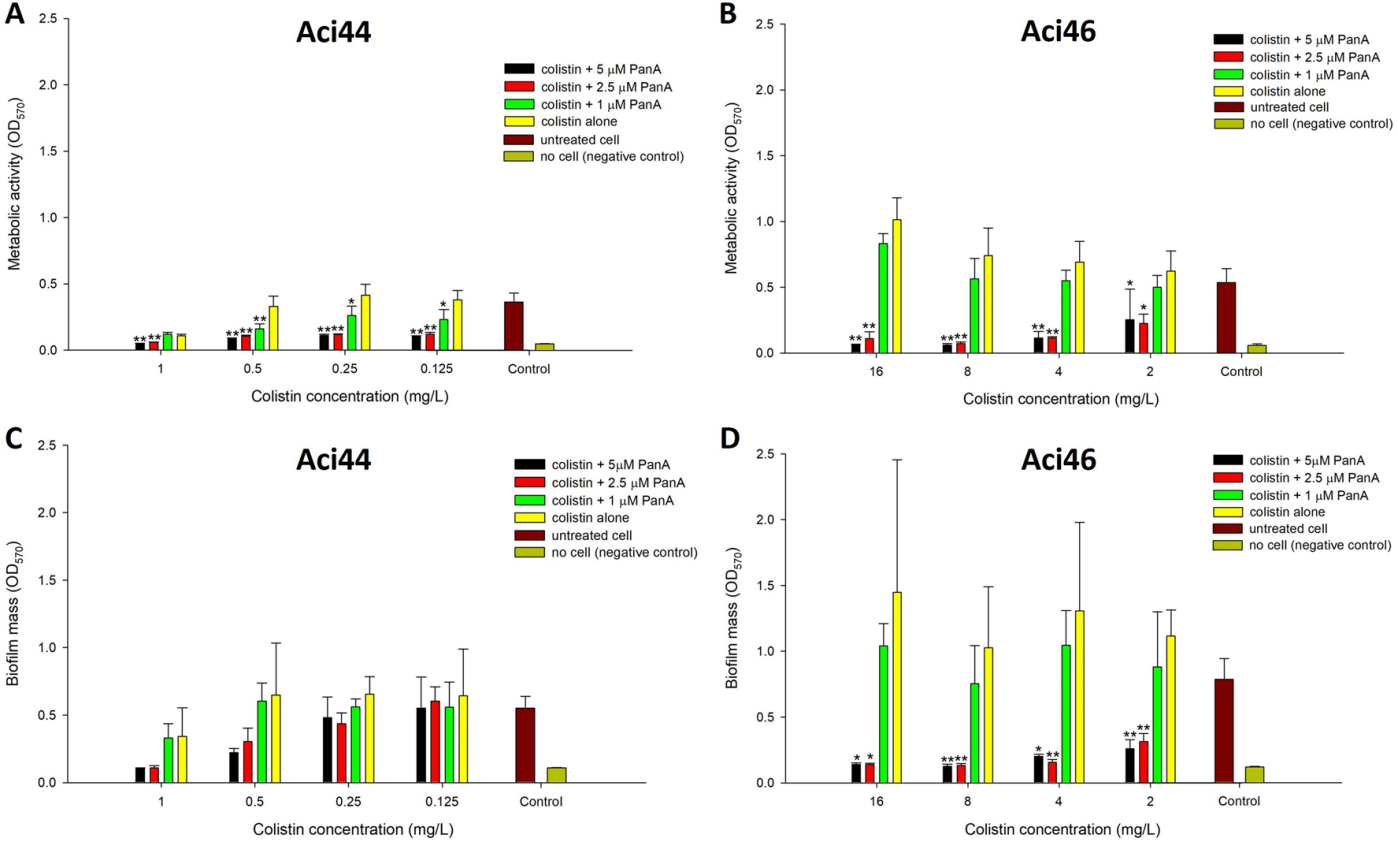
Anti-biofilm properties of combined panduratin A (PanA)-colistin compared with colistin alone against Aci44 (**A**, **C**) and Aci46 (**B**, **D**). (**A**, **B**) Metabolic activities of biofilm and (**B**, **C**) biofilm mass was determined using MTT staining and crystal violet, respectively. Black, red, green, brown, and dark yellow bars represent conditions of combined colistin with 5, 2.5, 1 µM PanA, untreated cell, and no cell (negative control), respectively, while yellow bar represents condition of colistin alone. The results are mean value with standard deviation as error bars three biological replicates. * and ** represent P < 0.05 and P < 0.01 compared with colistin alone, respectively.

One of the well-known virulence-associated characteristics of *A. baumannii* is its capability to form biofilm which can protect bacterial cells for against antibiotics^50^. Therefore, antibiotic agents that can eliminate *A. baumannii* in biofilm are excellent candidates for *A. baumannii* treatment. The present study determined whether the increase in sensitivity of colistin was associated with the anti-biofilm properties of panduratin A. The biofilm of *A. baumannii* was stained for metabolic activities. Panduratin A (1, 2.5, or 5 µM) in combination with four concentrations of colistin starting with 0.5xMIC in each strain (1 mg/L colistin for Aci44 and 16 mg/L colistin for Aci46) were tested. We found that combination of panduratin A (2.5 and 5 µM) with colistin significantly reduced metabolic activities in biofilm and biofilm mass (Fig. 2) in comparison to colistin alone. Panduratin A at 2.5 and 5 µM could reduce metabolic activities in biofilm even at less than 0.5xMIC of colistin (0.125 mg/L) colistin (Fig. 2A,B). In addition to metabolic activity of the bacteria in the biofilm, combination of colistin and panduratin A at 2.5 and 5 µM trend to reduce the mass of biofilm compared with colistin alone in Aci44, however, combination of colistin and panduratin A at 2.5 and 5 µM significantly reduce biofilm mass in Aci46 (Fig. 2C,D).

**Figure 3:**
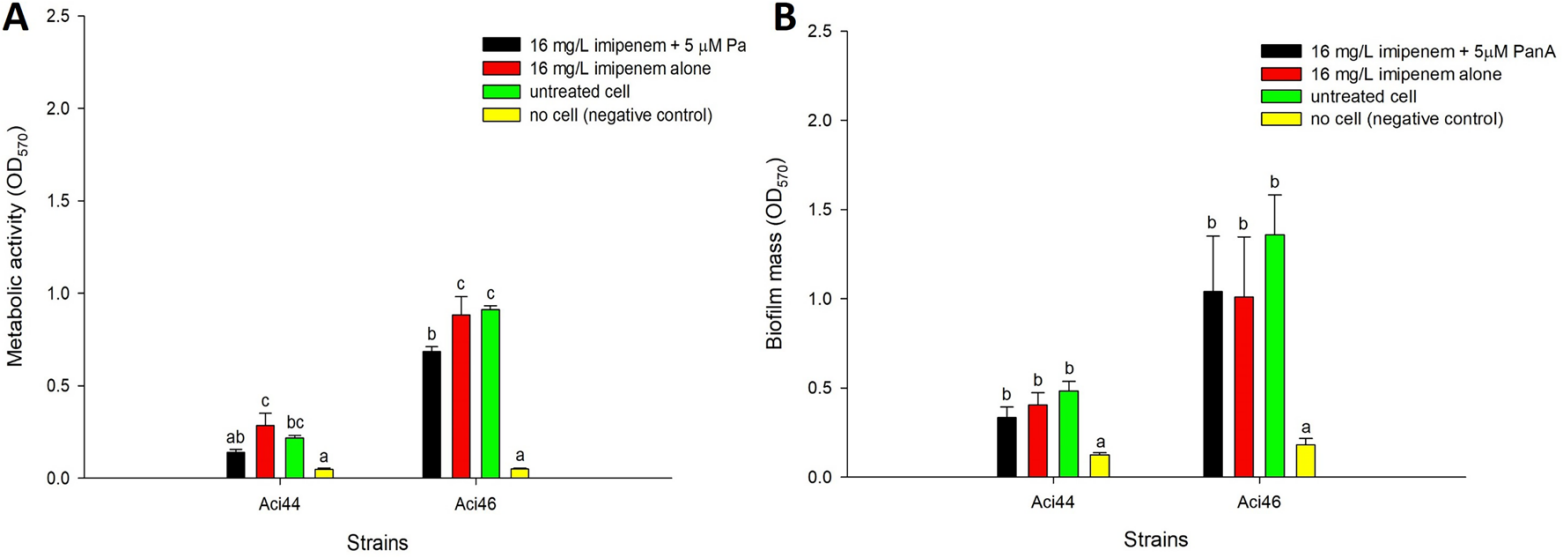
Anti-biofilm properties of panduratin A (PanA)-imipenem combinations against Aci44 and Aci46. Metabolic activities of biofilm were determined using MTT staining (**A**) and biomasses of biofilm were determined using crystal violet (**B**). Black, red, green, and yellow bars represent conditions of combined imipenem with 5 µM panduratin A, imipenem alone, untreated cell, and no cell (negative control), respectively. The results are mean values with standard deviations as error bars. Different letters (e.g., a and b) over error bars indicate statistically significant difference (*p* < 0.05) as determined by one-way ANOVA with Tukey post-hoc test. The experiments were done in three biological replicates.

We also tested whether panduratin A-imipenem showed the anti-biofilm properties in Aci44 and Aci46. The results showed that, indeed, panduratin A-imipenem combination could significantly reduce *A. baumannii* metabolic activities in biofilm whereas the combination did not reduce the biofilm masses in comparison to antibiotics alone (Fig. 3). Taken together, panduratin A can be used as an antibiotic adjuvant with colistin treatment, specifically for increasing bactericidal activity and enhancing anti-biofilm property.

### Complete genome of Aci44 and genomic analysis

Even though both Aci44 and Aci46 are colistin and carbapenem-resistant *A. baumannii*, the capacities of colistin resistance and response of panduratin A-antibiotic combination were different. As we have observed that Aci44 and Aci46 have different phenotypes (e.g., ability of biofilm formation and response to panduratin A-colistin combination), comparative genotypic characteristics among both strains might lend us insights on the different phenotypic features.

Genome data of Aci46 is available^8^ but Aci44 genome was not characterized. To investigate the genomic sequences of Aci44, short-read and long-read sequencing platforms were used and assembled to complete genome using hybrid assembly strategy. A summarized genome data of Aci44 is shown in Supplementary Table S1. Summary of comparison of antibiotic resistance and efflux pumps genes among Aci44 and Aci46 is shown in Supplementary Table S2. For instance, in Aci44, we found a partial-*pmrB* gene which is 861 bp and deduced to 286 amino acid residues (Supplementary Fig. S3), while full-length *pmrB* gene and deduced amino acids are 1344 bp and 444 residues, respectively^8,42^. Full results of comparative pangenome analysis are shown in Supplementary Tables S3–S6 and Fig. S4.

### Transcriptomic analysis

In order to investigate the mechanism or factor(s) that led to Aci44 cell death due to panduratin A-colistin combination, Aci44 transcriptomic profiles in various conditions were analyzed. Colistin at 0.05xMIC of panduratin A-colistin combination (0.1 mg/L colistin) was used. Aci44 was treated under four different conditions: CAMHB medium alone and medium with 5 µM panduratin A, 0.1 mg/L colistin, or 5 µM panduratin A + 0.1 mg/L colistin. A summary of RNA-Seq analyses is shown in Table 3. Total numbers of differentially expressed (DE) genes from panduratin A vs CAMHB, colistin vs CAMHB, panduratin A-colistin combination vs CAMHB, panduratin A vs colistin, panduratin A-colistin combination vs panduratin A, and panduratin A-colistin combination vs colistin comparisons were 21, 49, 50, 0, 114, and 240 genes, respectively. No DE genes were found from panduratin A vs colistin comparison. Panduratin A and colistin could stimulate the same pattern of gene expressions or systems.

**Table 3:** Summary of transcriptomic data from Aci44 under four different conditions.

Comparisons	Total differentially expressed genes	Upregulated genes	Downregulated genes
PanA vs CAMHB	21	3	18
Col vs CAMHB	49	30	19
Pcol vs CAMHB	50	28	22
PanA vs Col	0	0	0
Pcol vs PanA	114	63	51
Pcol vs Col	240	125	115

Col = colistin, PanA = panduratin A, Pcol = panduratin A-colistin combination.

**Figure 4:**
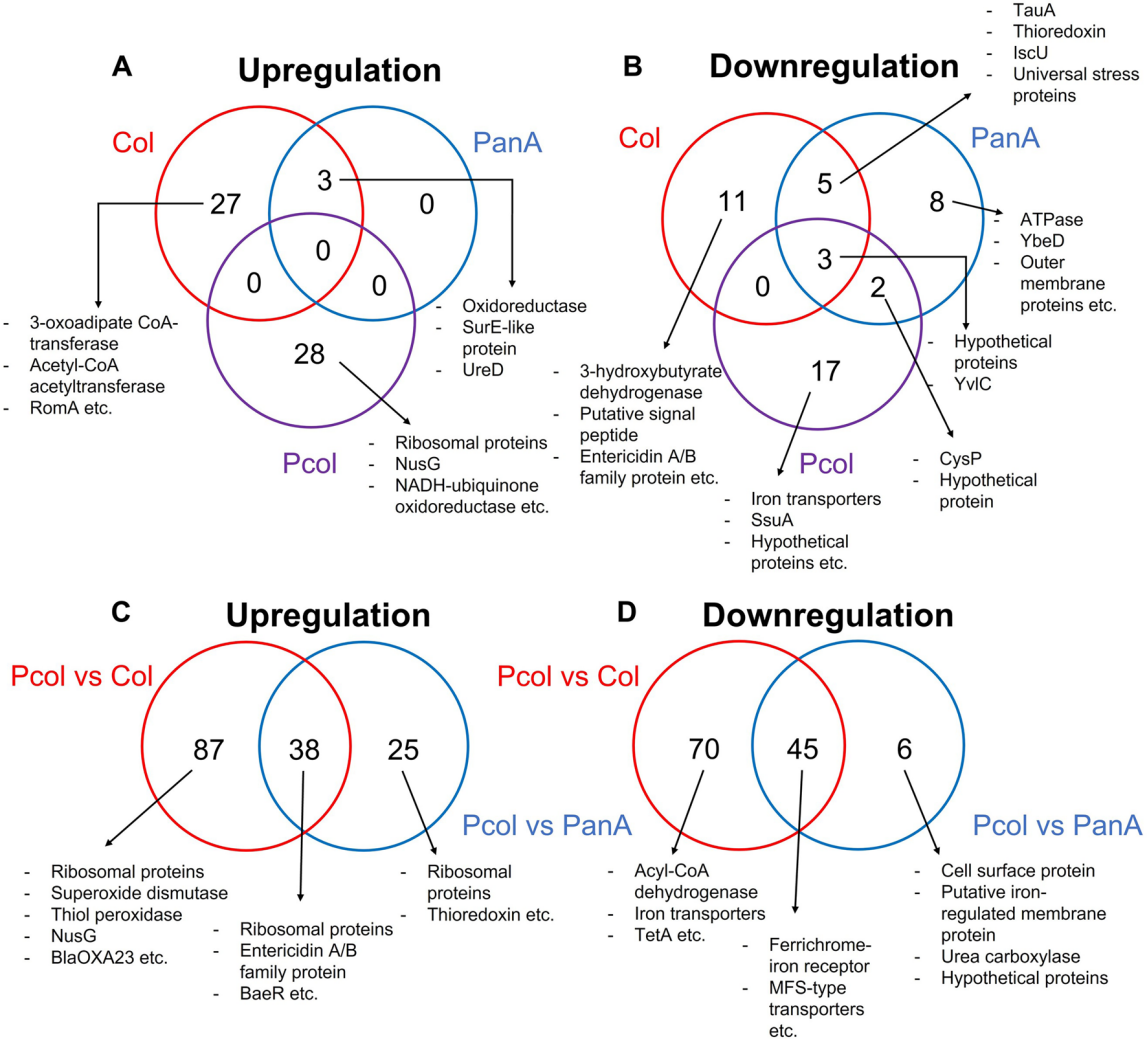
Venn-diagrams of unique and overlapped DE genes in Aci44 treated with single and combined compounds in comparison to CAMHB (**A**, **B**) and single compound in comparison to combined compound (**C**, **D**). Red, blue, and purple circles in (**A**) and (**B**) represent colistin (Col) vs CAMHB, panduratin A (PanA) vs CAMHB, and panduratin A-colistin combination (Pcol) vs CAMHB, respectively. Red and blue circles in (**C**) and (**D**) represent Pcol vs Col and Pcol vs PanA, respectively. The overlapped areas represent the shared DE genes with indicated numbers of genes among comparisons.

The unique and shared DE genes among comparisons are shown in Fig. 4 (fold change ≥ 4; Supplementary Tables S8 and S9). Exposures to single compounds (panduratin A or colistin) induced expression levels of the same set of genes i.e., genes encoding an oxidoreductase, SurE-like protein, and urase accessory protein UreD. All upregulated genes from panduratin A-colistin combination were not shared with panduratin A or colistin alone. Interestingly, eight encoding genes of ribosomal proteins were upregulated under panduratin A-colistin combination in comparison to CAMHB (Fig. 4A, Supplementary Table S8). All of these encoding genes of eight ribosomal proteins were further upregulated under panduratin A-colistin combination in comparison to both single compounds (panduratin A or colistin) (Fig. 4C, Supplementary Table S9). Genes involved in oxidative stress response (e.g., oxidoreductase, superoxide dismutase, thiol peroxidase) and ATP synthesis (e.g., NADH ubiquinone oxidoreductase, ATP synthase) were upregulated under panduratin A-colistin condition.

Three shared downregulated genes from the conditions of panduratin A, colistin, and panduratin A-colistin combination were genes encoding uncharacterized membrane protein YvlC and two hypothetical proteins (Fig. 4B). Iron-related genes were downregulated under exposure to single compounds or panduratin A-colistin combination. For instance, iron-sulfur cluster assembly scaffold protein IscU gene was downregulated under colistin or panduratin A exposure (Fig. 4B) while iron transporter genes were downregulated under panduratin A-colistin combination. In addition, ferrichrome-iron receptor gene was downregulated in panduratin A-colistin in comparison to single compound conditions (Fig. 4D). Iron homeostasis of Aci44 might be disrupted by panduratin A-colistin combination. Panduratin A also mediated expression levels of two antibiotic resistance genes, carbapenemase encoding *bla*_OXA-23_ and major facilitator superfamily (MFS)-type transporter for tetracycline resistance encoding *tetA*. The *bla*_OXA-23_ was upregulated while *tetA* was downregulated expression (Fig. 4C,D). Moreover, other MFS-type transporters were downregulated. Reducing expression of MFS-type transporters could facilitate ROS and colistin accumulation.

### Panduratin A-colistin combination increases ROS production

**Figure 5:**
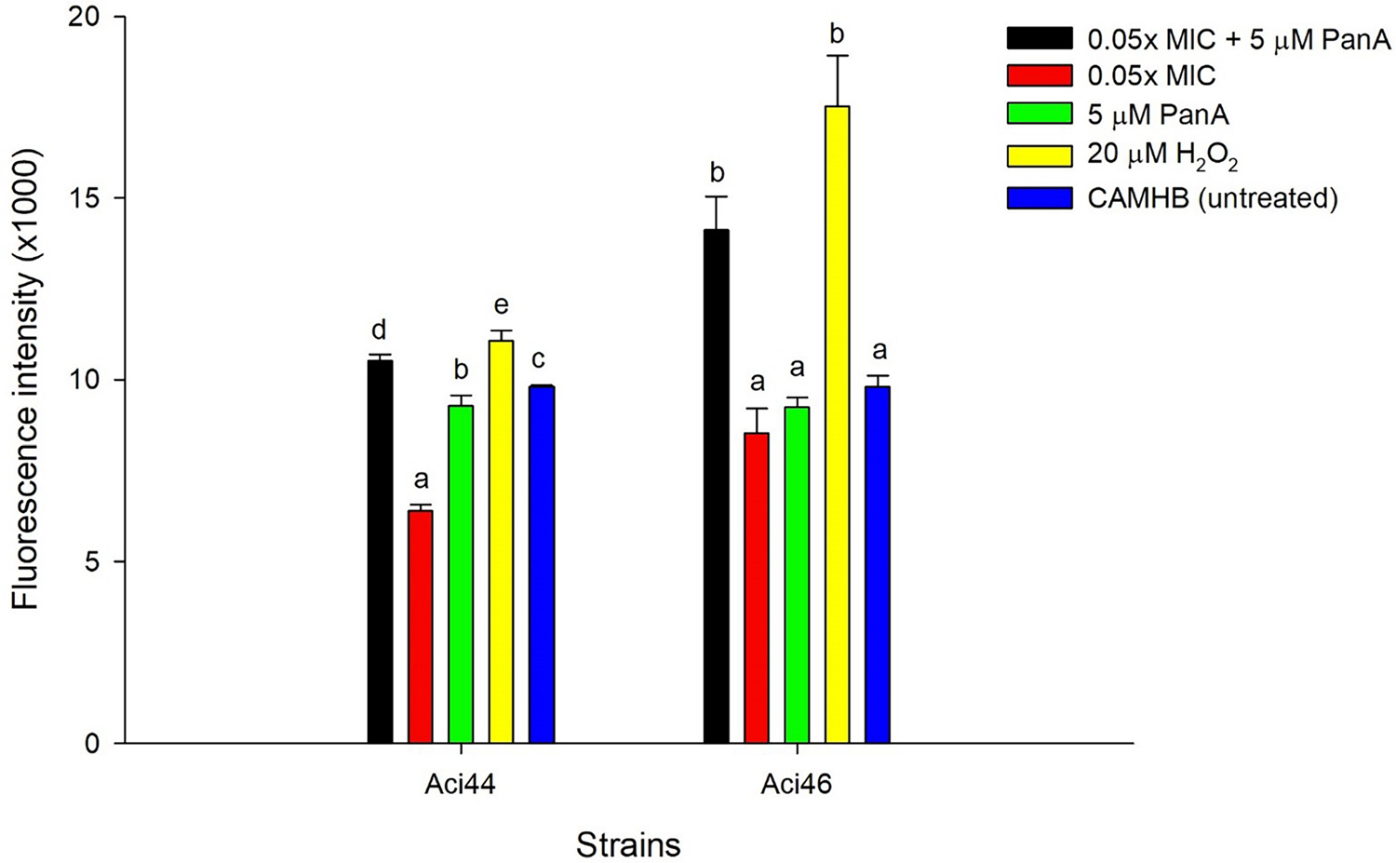
ROS production in Aci44 and Aci46 cells treated with single and combined compounds. The black, red, green, yellow, and blue represent 0.05 × MIC colistin combined with 5 µM panduratin A, 0.05 × MIC colistin, 5 µM panduratin A, 20 µM H_2_O_2_, and CAMHB, respectively. The results are mean value with standard deviation as error bars. Different letters (e.g., a and b) over error bars indicate statistically significant difference (*p* < 0.05) as determined by one-way ANOVA with Tukey post-hoc test. The experiments were done in three biological replicates.

Based on our RNA-Seq results, it appears that the panduratin A-colistin combination may lead to an increase in reactive oxygen species (ROS) production. To validate this finding, we conducted ROS production assays on treated strains Aci44 and Aci46 using combinations of colistin and panduratin A, as well as individual treatments with colistin alone and panduratin A alone (Fig. 5). H_2_O_2_ and media were used as controls. Our findings indicated that the panduratin A-colistin combination resulted in a significant increase in ROS production in both Aci44 and Aci46 strains compared to either treatment alone. This suggests an enhancing effect of the combination treatment on ROS production, potentially enhancing the antimicrobial activity against these strains.

## Discussion

The urge to discover new drugs against multidrug resistance bacteria are inevitably pressing. Combination therapy becomes one of alternative therapeutic methods to treat severe multidrug resistance bacterial infection^51^. Natural compounds are attractive choices to explore for their capacities as antimicrobials and/or adjuvants^9^. In this study, panduratin A was chosen and has been shown to be an excellent colistin adjuvant. Panduratin A-colistin combination can kill colistin- and carbapenem-resistant *A. baumannii* Aci44 and Aci46, although such enhancement was not observed with panduratin A-imipenem combination. The differences in adjuvant properties with colistin and imipenem could lie in the differences in the modes of action. One of the possible mechanisms in markedly enhancing the bactericidal activities of colistin is that panduratin A could facilitate colistin access the lipid A molecule on the bacterial cell wall which has been reported as colistin’s target^5^. We then further hypothesized that, in combination treatment, panduratin A could clear biofilm which is one of the bacterial extracellular matrices and allowing better antibacterial activity of colistin. This hypothesis is supported by our results showing panduratin A-colistin combination could reduce biofilm mass and metabolic activity in the biofilm. Presence of biofilm masses might shield entry of imipenem into the bacteria and prevent the targets of imipenem.

As Aci44 and Aci46 showed different responses to single and combined compounds, genome analyses were applied in an attempt to explain the different phenotypic results. We found that *pmrB* from Aci44 and Aci46 were different. The *pmrB* gene encodes polymyxin resistant component PmrB of the PmrAB two-component system and, as a result, confers colistin resistance characteristics^52^. Incomplete-PmrB in Aci44 might affect or reduce colistin resistance when compared to Aci46. In addition, based on SNP analysis in comparison to ATCC 17978, PmrB from Aci46 have three non-synonymous mutations: Ala138Thr, Pro200Leu, and Glu229Asp that might affect or enhance colistin resistance characteristics in Aci46^8^. Additionally, we compared genome data of CCRAB (i.e., Aci44 and Aci46) and ATCC 17978 in order to identify the cause of phenotypic variations in biofilm and lipopolysaccharide (LPS) formation. We identified mutations in genes of *pgaABCD* operon and lipid A synthesis genes. The *pgaA, pgaB,* and *pgaC* are parts of the *pgaABCD* operon that encodes proteins for cell-associated PGA, one of most essential biofilm structures in *A. baumannii*^50,53^. In lipid A synthesis, LpxL catalyzes transfer of a laurate group from an acyl carrier protein (ACP) onto the R-2′-hydroxymyristate acyl chain of Kdo2-lipid IV_A_^54^. Lipid A phosphoethanolamine transferase is an intramembrane enzyme which modifies the lipid A portion of LPS by adding phosphoethanolamine, this modification affects net-negative charge of outer membrane and resistance to polymyxin^55^. MsbA is an important ATP-binding cassette that mediates transfer of major lipids (e.g., phospholipids, lipopolysaccharides) from the cytoplasm to the periplasm^56^. MsbA was also classified as a potential drug target on LPS^57^. These identified SNPs might involve capacity of biofilm formation and lipid A biosynthesis in CCRAB, especially lipid A phosphoethanolamine transferase and MsbA which directly link to drug resistance property. When we compared the genomes of Aci44 and Aci46, unique point mutations in lipid A synthesis were identified in Aci46, specifically in D-arabinose-5-phosphate isomerase encoding gene and in lipid A disaccharide synthase LpxB encoding gene. D-arabionse-5-phosphate isomerase is the enzyme which regulates reversible isomerization of D-ribulose 5-phosphate (Ru5P) to D-arabinose-5-phosphate, a precursor of 3-deoxy-D-manno-octulosonate (KDO) in LPS formation^58^. Lipid-A-disaccharide synthase LpxB is a glycosyltransferase in the lipid A synthesis pathway that catalyzes nucleophilic attack on 6′-hydroxyl of lipid X on the anomeric carbon of UDP-diacyl-glucosamine (UDP-DAG) to produce β(1-6)-tetraacyl-disaccharide 1-phosphate (lipid A disaccharide) and UDP as a leaving group^59^. These mutations in lipid A biosynthesis genes could result in different lipid A and LPS production or levels in Aci44 and Aci46 affecting varying phenotypes when exposed to colistin and panduratin A -colistin combination.

Mechanisms of action of panduratin A-colistin combination were studied by RNA-Seq analyses in Aci44. We found that panduratin A-colistin combination induces expression of ribosomal proteins, oxidative phosphorylation during respiration, and ROS production while iron transporter and MFS-type transporter systems were suppressed. Based on transcriptomic analysis, ROS could be generated by colistin, induced expression of ribosomal proteins and proteins in electron transport chain, and dysregulation of iron uptake. Colistin induces hydroxyl radical production as alternative mechanism^60,61^. The ribosomal proteins could induce intracellular ROS by an unknown mechanism^62^. Disturbance of iron homeostasis (e.g., low ferric [Fe^3+^] iron) prevents autophosphorylation of sensing kinase PmrB and phosphoryl group transfer to response regulator PmrA, which prevents *arnT* and *eptA* expression and eventually lipid A modification^63^. Incomplete PmrB in Aci44, as shown by our genome data, means lipid A modification with phosphoethanolamine could be further impaired. In addition, high ferrous (Fe^2+^) iron can promote ROS production via Fenton chemistry^64^. The accumulation of ferrous iron and ROS cause cell ferroptosis^65^. A recent study has shown that natural flavonoids (i.e., 7,8-dihydroxyflavone, myricetin, and luteolin) could enhance colistin efficacy by changing the iron form from ferric to ferrous and the accumulation of ferrous form is able to buildup ROS^64^. Panduratin A might enhance colistin potency in the same mode of action with flavonoids.

## Conclusion

Panduratin A-colistin combination could be an alternative choice for eliminating colistin-resistant *A. baumannii*. The combination works against colistin- and carbapenem-resistant *A. baumannii* Aci44 and Aci46 with bactericidal and anti-biofilm properties. Sharing genotypic characteristics in Aci44 and Aci46 could be specific features for colistin- and carbapenem resistance in *A. baumannii*. Based on Aci44 genome analysis, partial *pmrB* and deletions on encoding genes for biofilm and lipid A biosyntheses could be the factors to reduce colistin resistance potential in Aci44. These mutations should be studied for their functional effects on drug resistance activities as they might be new markers of colistin- and carbapenem-resistant *A. baumannii*. The mode of panduratin A-colistin combination action in Aci44 is studied by using transcriptomic analysis. We found that encoding genes for ribosomal proteins, and oxidative stress response were upregulated while iron transport and MFS-type transporters were downregulated. These could be led to ROS accumulation and bacterial ferroptosis. Since panduratin A shows an effectiveness at low concentration (5 µM), it is considered as a good adjuvant candidate for treatment of colistin resistance *A. baumannii*. To ensure, adjuvant potency, future studies using panduratin A in combination with colistin in animal model are required.

### Supplementary Information

Supplementary Information.

Supplementary Tables.

## Data Availability

Complete genome of *A. baumannii* Aci44 and plasmid sequence of pAci44a have been deposited at DDBJ/ENA/GenBank under accession number CP101653 and CP101654, respectively. RNA-Seq data have been deposited at the same database under BioProject number PRJNA963471, PRJNA964451, PRJNA966166, and PRJNA967317 for CAMHB, 0.1 mg/L colistin, 5 µM panduratin A, and combined 0.1 mg/L colistin, and 5 µM panduratin A, respectively (accession number shown in Table S7).
