# Supplementary material for: Enhancing effect of natural adjuvant, panduratin A, on antibacterial activity of colistin against multidrug-resistant Acinetobacter baumannii
Source: Sci Rep. 2024 Apr 29;14:9863. doi: 10.1038/s41598-024-60627-0 (PMC11059350; doi:10.1038/s41598-024-60627-0)
Supplement: Supplementary file 1 — Supplementary Information. [file 41598_2024_60627_MOESM1_ESM.docx]

**Supplementary data**

Synergistic effect of natural adjuvant, panduratin A, on antibacterial activity of colistin against multidrug-resistant *Acinetobacter baumannii*

**Results**


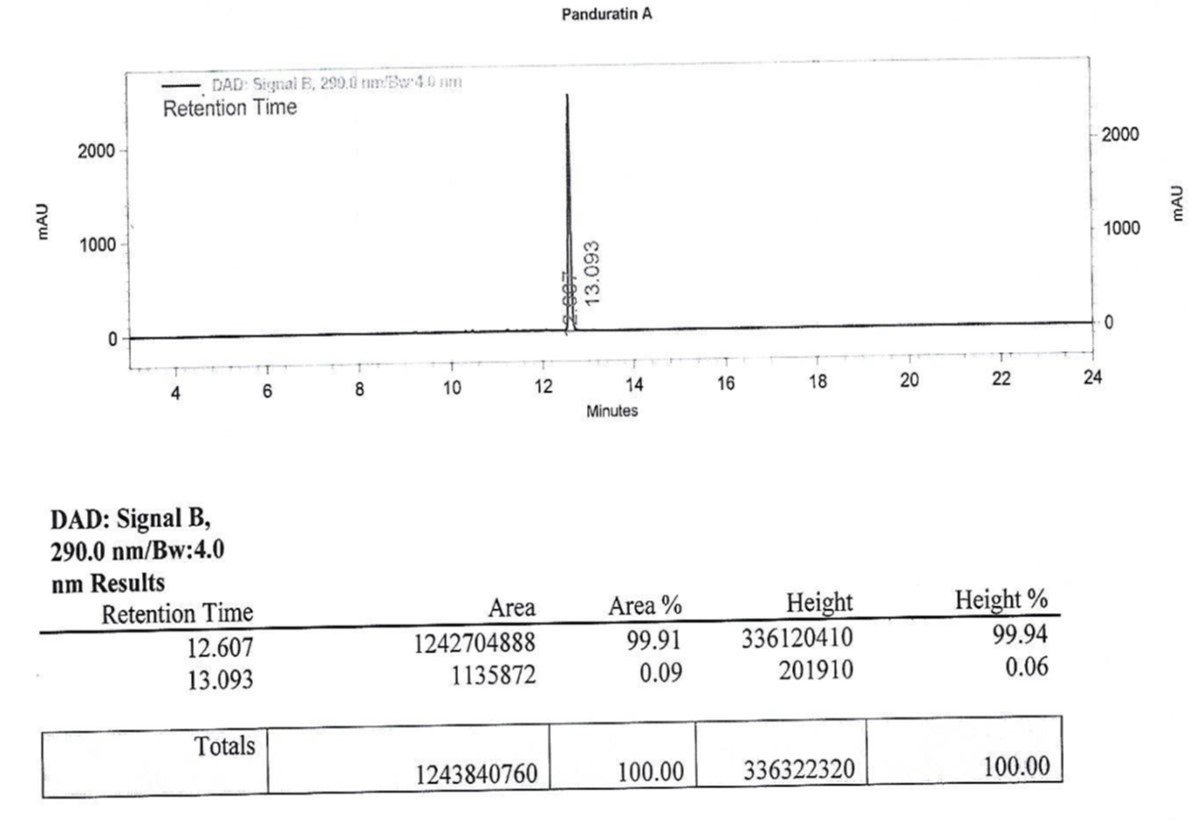


**Figure S1** Determination of panduratin A purity by HPLC.


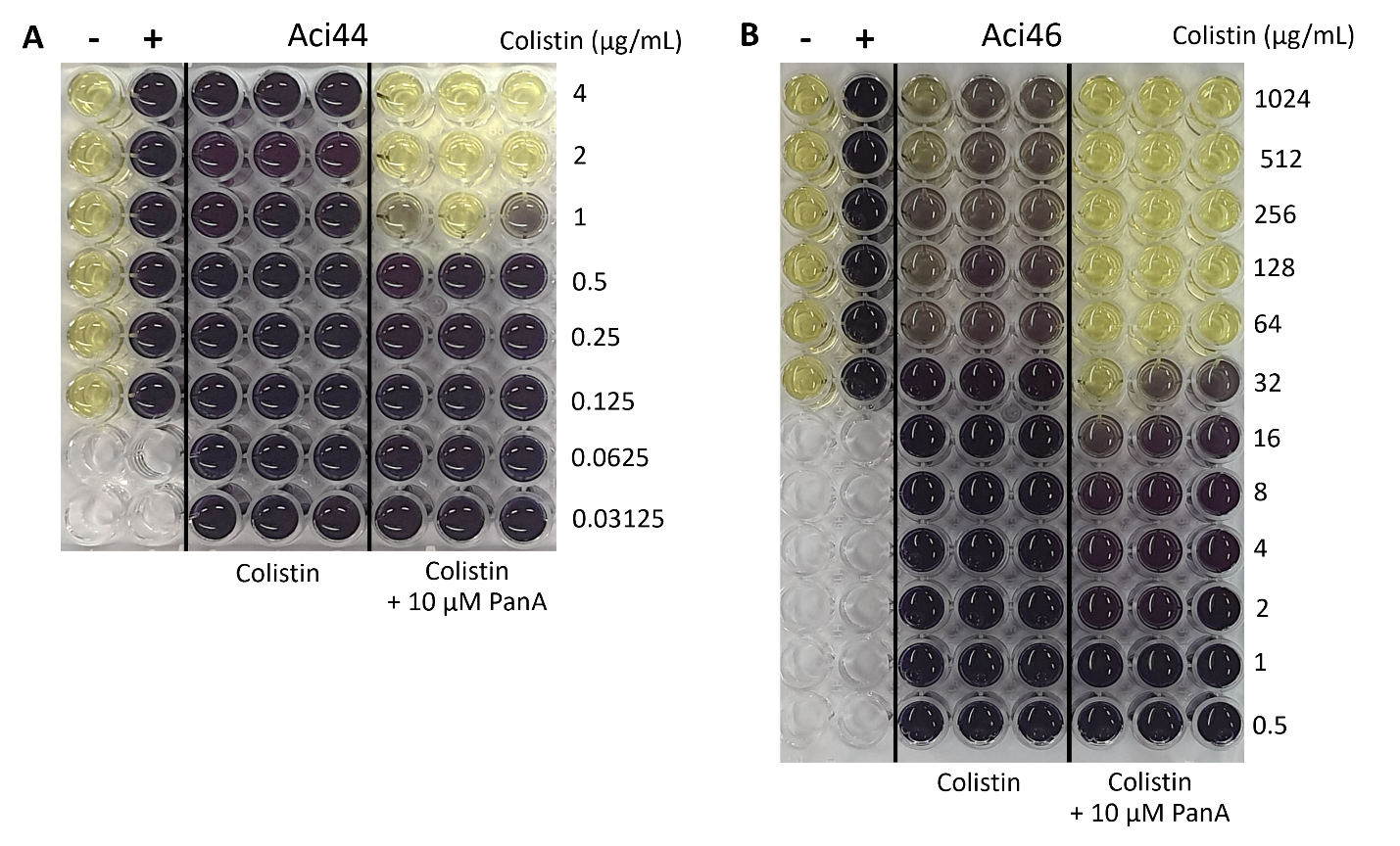


**Figure S2** MIC determination of combination of 10 µM PanA and colistin, and colistin alone, by microdilution assay using MTT staining. Combined compounds and single compound treated Aci44 (A) and Aci46 (B). Viable cell and positive control showed in purple while dead cell and negative control showed in yellow. The experiments were tested in three individual replicates.

## Complete genome of Aci44 and antibiotic resistance gene prediction

Even though both Aci44 and Aci46 are colistin and carbapenem-resistant *A. baumannii*, the capacities of colistin resistance and response of panduratin A-antibiotic combination were different. Comparative genotypic characteristics among both strains might give us the explanation for different phenotypic features. Genome data of Aci46 is available (1) but Aci44 genome was not characterized. To investigate the genomic sequences of Aci44, short-read and long-read sequencing platforms were used and assembled to complete genome using hybrid assembly strategy. A summarized genome data of Aci44 is shown in Table S7. The chromosome of Aci44 is 3,974,434 bp in length, with a G + C content of 39.01%. We identified one 8,731 bp plasmid, named pAci44a with a G + C content of 34.37%. No antibiotic resistance genes were found on the plasmid. Results of identification of Aci44 using ribosomal MLST confirmed that Aci44 is *A. baumannii* at 100% identity based on variation of 54 encoding genes for ribosomal protein subunits.

Summary of comparison of antibiotic resistance and efflux pumps genes among Aci44 and Aci46 is shown in Table S8. We found that antibiotic resistance and efflux pump gene predictions in Aci44 are like those of Aci46. Aci44 carries *aph(3')-Ia* and *bla*_TEM-1_ genes while Aci46 harbors *sul2* and *pmrB* genes. In Aci44, we found a partial-*pmrB* gene which is 861 bp and deduced to 286 amino acid residues (Figure S3), while full-length *pmrB* gene and deduced amino acids are 1,344 bp and 444 residues, respectively (1, 2). The partial-PmrB from Aci44 showed 95.10% and 95.45% amino acid sequence similarity to PmrB from Aci46 and ATCC 17978, respectively.


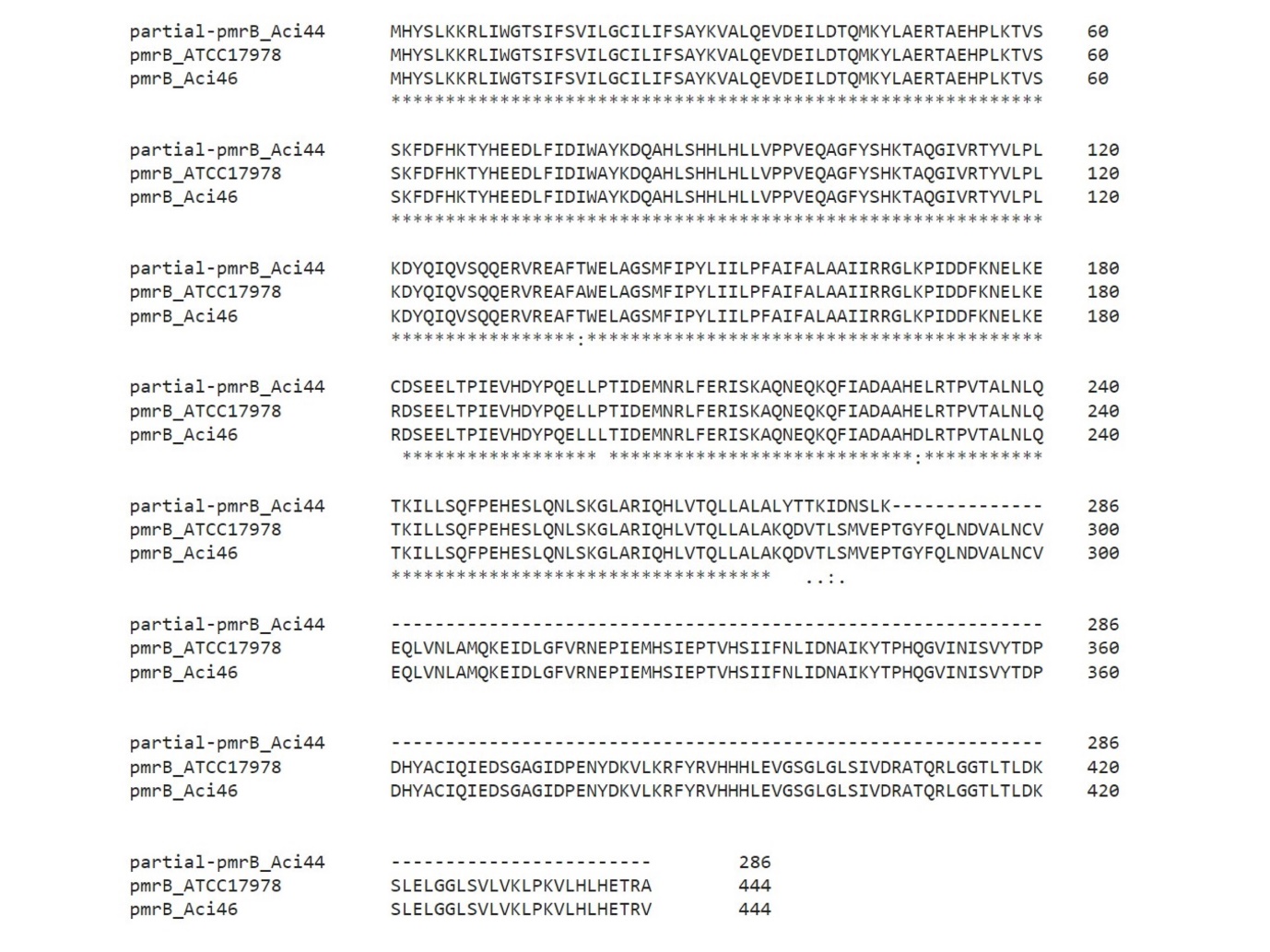


**Figure S3** Deduced amino acid sequence alignment among full length PmrB’s from Aci46 and ATCC 17918 and a partial PmrB from Aci44.

## Pangenome comparison and pairwise SNP analysis

We have observed that Aci44 and Aci46 have different phenotypes (e.g., ability of biofilm formation and response to panduratin A-colistin combination) and genotypes. We further investigate biofilm formation and lipid A biosynthesis among strains which might explain the phenotypic variations. The comparison of Aci44, Aci46, and ATCC 17978 (drug sensitive) genomes was analyzed to identify to unique or shared features. Results of comparative pangenome analysis are shown in Figure S4. Pangenome (all genes and all strains) and core genes (present in all strains) comprise 4,155 and 2,793 genes, respectively (Table S1). Strain-specific genes for Aci44, Aci46, and ATCC 17978 are 156, 142, and 449 genes, respectively (Table S2). Shared features between Aci44 and Aci46 (renamed CCRAB-specific) are 566 genes (Table S3). Several encoding genes for antibiotic resistance are in CCRAB-specific genes, for example, *bla*_OXA-23_*, msr(E), aph(3”)-Ib, aph(6)-Id, mph(E), tetA,* and *tetR*. The *czcA, czcB*, and *czcC* that encode CzcCBA cobalt/zinc/cadmium efflux RND transporter are also CCRAB-specific. CzcCBA cobalt/zinc/cadmium efflux RND transporter is a channel for exporting cations (3) and involves with heavy metal resistance (4). This efflux pump is consistently found in polymyxin and carbapenem-resistant *A. baumannii* (Aci46, AC30, and R14) (1) and associated with extensively drug resistance characteristics (5).


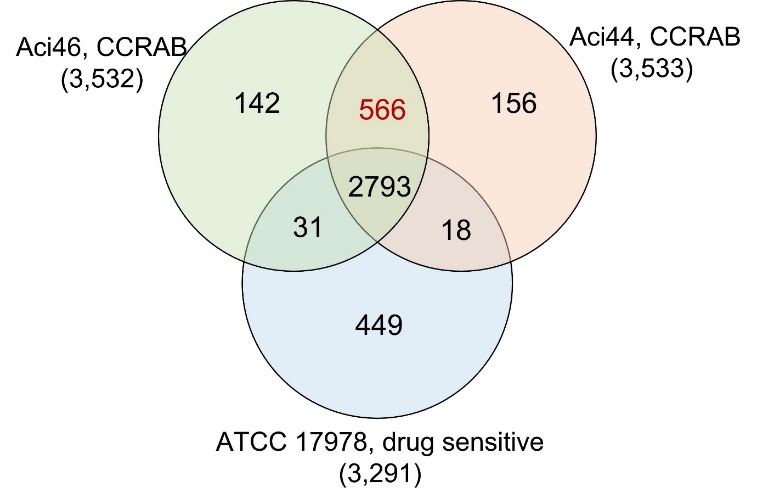


**Figure S4** Venn diagram of comparative pangenome analysis among *A. baumannii* isolates. Green, orange, and blue areas represent Aci46, Aci44, and ATCC 17978, respectively, with numbers of total genes. The central area shows encoding core genes presented in all strains. The overlapped areas show numbers of encoding genes shared among strains. The red number represents encoding genes shared between Aci44 and Aci46. CCRAB: colistin and carbapenem-resistant *A. baumannii*.

Known genes for biofilm formation and lipid A biosynthesis are among core genes. To further explore the associated mutations in encoding genes of biofilm and lipid A processing from Aci44 and Aci46, pairwise single nucleotide polymorphism (SNP) analysis was used to detect non-synonymous SNP, insertion, and deletion points. We found that Aci44 and Aci46 shared characters of non-synonymous SNPs in three genes in biofilm biosynthesis and three genes in lipid A biosynthesis, when compared with ATCC 17978 (Table S9). Three genes in biofilm biosynthesis that encode biofilm poly-beta-1,6-N-acetyl-D-glucosamine (beta-1,6-GlcNAc; PGA) outer membrane secretin PgaA, biofilm PGA synthesis deacetylase PgaB, and biofilm PGA synthesis N-glycosyltransferase PgaC showed sharing non-synonymous SNPs, however, *pgaA* and *pgaC* genes showed nucleotide deletions and frameshift. Mutations in biofilm biosynthesis genes from Aci44 consist of A, AA, and A deletions in *pgaA* at nucleotide residues 211, 1,286 – 1,287, and 1,375, respectively, and T deletion in *pgaC* at nucleotide residue 890. Three genes in lipid A biosynthesis contained with SNPs in genes encoding lipid A biosynthesis lauryl acyltransferase (LpxL), putative lipid A phosphoethanolamine transferase, and lipid A export permease/ATP-binding protein MsbA. Nucleotide deletions and frameshift appeared in four genes encoding gene of D-arabinose-5-phosphate isomerase (i.e., A deletion at nucleotide residue 886), lipid A biosynthesis lauryl acyltransferase (i.e., A deletion at nucleotide residue 336), putative lipid A phosphoethanolamine transferase (i.e., TA, T, and T deletions at nucleotide residues 111 – 112, 351, and 405, respectively), and lipid-A-disaccharide synthase LpxB (i.e., T deletion at nucleotide residue 463). These deletions might affect phenotypic differences between Aci44 and Aci46 i.e., panduratin A effect on biofilm formation, lipid A modification, and higher colistin resistance in Aci46.

References

1. Thadtapong N, Chaturongakul S, Soodvilai S, Dubbs P. Colistin and Carbapenem-Resistant Acinetobacter baumannii Aci46 in Thailand: Genome Analysis and Antibiotic Resistance Profiling. Antibiotics (Basel). 2021;10(9).

2. Smith MG, Gianoulis TA, Pukatzki S, Mekalanos JJ, Ornston LN, Gerstein M, et al. New insights into Acinetobacter baumannii pathogenesis revealed by high-density pyrosequencing and transposon mutagenesis. Genes Dev. 2007;21(5):601-14.

3. Nies DH. The cobalt, zinc, and cadmium efflux system CzcABC from Alcaligenes eutrophus functions as a cation-proton antiporter in Escherichia coli. J Bacteriol. 1995;177(10):2707-12.

4. Nies DH. Efflux-mediated heavy metal resistance in prokaryotes. FEMS Microbiology Reviews. 2003;27(2-3):313-39.

5. Gheorghe I, Barbu IC, Surleac M, Sarbu I, Popa LI, Paraschiv S, et al. Subtypes, resistance and virulence platforms in extended-drug resistant Acinetobacter baumannii Romanian isolates. Sci Rep. 2021;11(1):13288.
